# Loss of NFBD1/MDC1 disrupts homologous recombination repair and sensitizes nasopharyngeal carcinoma cells to PARP inhibitors

**DOI:** 10.1186/s12929-019-0507-z

**Published:** 2019-02-04

**Authors:** Zhihai Wang, Wenqi Zuo, Quan Zeng, Yi Qian, Yanshi Li, Chuan Liu, Jue Wang, Shixun Zhong, Youquan Bu, Guohua Hu

**Affiliations:** 1 Department of Otorhinolaryngology, the First Affiliated Hospital of Chongqing Medical University, Chongqing, 400016 China; 2 Department of Biochemistry and Molecular Biology, Molecular Medicine and Cancer Research Center, Chongqing Medical University, Chongqing, 400016 China

**Keywords:** Nasopharyngeal carcinoma, PARP inhibitor; homologous recombination, NFBD1/MDC1, DNA damage response

## Abstract

**Background:**

Nasopharyngeal carcinoma (NPC), a highly invasive tumor, exhibits a distinctive racial and geographic distribution. As options of agents for effective combination chemoradiotherapy for advanced NPC are limited, novel therapeutic approaches are desperately needed. Here the potential of silencing NFBD1 in combination with PARP inhibition as a novel therapeutic strategy for NPC was investigated.

**Methods:**

To investigate the function of NFBD1, we created NFBD1-depleted NPC cell lines via lentivirus mediated shRNA, and the colony formation, MTS assay, comet assay and apoptosis analysis were used to evaluate the sensitivity of NFBD1 knockdown on PARP inhibition. The signaling change was assessed by western blot, Immunofluorescence and flow cytometry. Furthermore, Xenografts model was used to evaluate the role of silencing NFBD1 in combination with PARP inhibition.

**Results:**

We find that silencing NFBD1 in combination with PARP inhibition significantly inhibits the cell proliferation and cell cycle checkpoint activity, and increases the apoptosis and DNA damage. Mechanistic studies reveal that NFBD1 loss blocks olaparib-induced homologous recombination repair by decreasing the formation of BRCA1, BRCA2 and RAD51 foci. Furthermore, the xenograft tumor model demonstrated significantly increases sensitivity towards PARP inhibition under NFBD1 deficiency.

**Conclusions:**

We show that NFBD1 depletion may possess sensitizing effects of PARP inhibitor, and consequently offers novel therapeutic options for a significant subset of patients.

## Background

Nasopharyngeal carcinoma (NPC), a highly invasive cancer, is a common highly malignant head and neck cancer derived from the epithelium of nasopharynx. It is prevalent in Southern China, Malaysia, and Singapore [1, 2]. Although technical improvements in diagnostic technology and clinical treatment, including radiotherapy and chemotherapy, local recurrences and distant metastasis often occur in 30–40% of NPC patients at advanced staged, and majority of patients will also ultimately die of their disease [3].

Poly (ADP-ribose) polymerase (PARP) is a nuclear enzyme that senses DNA single strand breaks (SSBs). When PARP is inhibited, SSBs are converted into double-strand DNA breaks (DSBs) through collapse of the replication fork. DSBs can be repaired by homologous recombination (HR) which is a high fidelity, error-free form of DNA repair [4]. BRCA1 and BRCA2 proteins are critical components in the process of homologous recombination repair (HRR) for the repair of DSBs, in BRCA-deficient tumors, HRR is not functional, and therefore the cell is hypersensitive to PARP inhibitors [5–7]. However, PARP inhibitors could also potentially be used as agents that enhance chemo- or radiotherapy-induced DNA damage in patients without defined gene mutations [8]. Therefore, the other mutations/deletions in DNA damage repair genes which have been used to enhance the sensitivity of PARP inhibitors have being widely investigated.

NFBD1 (also known as KIAA01770 or MDC1) is an identified nuclear protein that regulates many aspects of the DNA damage-response pathway, such as intra-S phase checkpoint, G2/M checkpoint, and spindle assembly checkpoint [9–11]. Human NFBD1 comprises 2089 amino acid residues, has a predicted molecular weight of ~ 220 kDa, and contains an FHA (Forkhead Associated) domain two BRCT (BRCA1 carboxy terminal) domains [12]. These are important structures shared by many DNA damage response proteins, such as Chk2, NBS1 and the tumor suppressor BRCA1. Recent studies have shown that NFBD1 is a participant in the early response to DNA damage and its subsequent signaling within cells. NFBD1 exists in a complex with Chk2 and BRCA1 [9, 13], which are proteins involved in the pathway of homologous recombination. Furthermore, the observed nuclear colocalization of NFBD1 with BRCA1 is further suggestive of a role for NFBD1 in homologous recombination. We focused on NFBD1 in this study and showed that NPC cells with NFBD1-deficient are hypersensitive to the PARP inhibitors olaparib. Thus, PARP inhibitors have therapeutic potential in the treatment of NFBD1-defcient NPC, and our results might extend the concept of synthetic lethality to tumors bearing alterations in NFBD1.

## Methods

### Cell lines and reagents

CNE1, CNE2 and HNE1 were obtained from the Molecular Medicine and Cancer Research Center, Chongqing Medical University. The cells were grown in RMPI-1640 medium (HyClone, Logan City, Utah, USA) with 10% fetal bovine serum (HyClone, Logan City, Utah, USA) at 37 °C with 5% CO_2_. The lentivirus-mediated shNFBD1 and shControl were purchased from Genechem, Shanghai,

China. PARP inhibitor Olaparib (AZD2281) was obtained from MedChemExpress (Princeton, NJ, USA). Hoechst 33342 were purchased from Beyotime Institute of Biotechnology (Nantong, China).The antibodies used in this study were anti-NFBD1 (Abcam, UK); anti-RAD51, anti-BRCA1, anti-BRCA2, and anti-PARP1 (Santa Cruz Biotechnology, USA); anti-γ-H2AX (Cell Signaling Technology, Danvers, MA, USA).

### Lentivirus infection

The lentiviral transduction was performed as previously described [11, 14–16]. Cells were transferred into six-well plates, and then viral supernatants were added. The transfected cells of stable expression shNFBD1 and shControl were obtained under puromycin (1 μg/ml).

### RNA extraction and real-time quantitative RT-PCR (qRT-PCR)

Total cellular RNA was extracted using TRIzol reagent (Invitrogen, Carlsbad, CA, USA) according to the manufacturer’s instructions. One microgram of total RNA was used to synthesize cDNA using the One-Step SYBR PrimeScriptTM RT-PCR Kit II (TaKaRa Biotechnology, Dalian, China). The qRT-PCR was performed using SYBR Premix Ex Taq in a LightCycler 480 qRT-PCR system (Bio-Rad, Hercules, CA, USA). The qRT-PCR primers of NFBD1 were NFBD1-F (AGCAACCCCAGTTGTCATTC) and NFBD1-R (TCCACCACCCTGTTGCTGTA). The 2^-ΔΔCt^ method was used to determine the relative quantification of NFBD1 expression.

### Clonogenic survival and cytotoxicity assays

For clonogenic survival assay, 500 cells were seeded in 6-well plates in triplicate. After 24 h, the cells were treated continuously with various concentrations of olaparib for another 24 h. After 10–14 days, colonies were fixed with methanol, and stained with crystal violet solution. Colonies containing 50 or more cells were counted as survivors. For cytotoxicity assays, cells were seeded in 96 well plates, and subjected to olaparib treatment as indicated for 48 h. Cell growth was examined using the Promega MTS assay [14–16].

### Flow cytometry

Cells were exposed to olaparib or vehicle for 24 h. For cell cycle analysis, cells were washed in PBS, fixed in 70% ice-cold ethanol at 4 °C overnight and stained with 50 μg/ml propidium iodide (PI) solution containing 0.2% Triton X-100 and 100 μg/ml DNase-free RNase A. For apoptosis analysis, cells were harvested and stained using Annexin V-FITC Apoptosis Detection kit (Beyotime Institute of Biotechnology, Nantong, China) and according to manufacturer’s recommendation. For γ-H2AX, RAD51 and G2/M checkpoint analysis, cells were fixed with ethanol, re-suspended in PBS containing 0.25% (vol/vol) Triton X-100, incubated on ice for 15 min and then incubated in γ-H2AX, RAD51 or phospho-histone H3 (Ser10) antibody for 1 h at room temperature. Samples were then incubated for 30 min at room temperature with secondary antibodies and were determined analyzed by a FACSVantage SE system (BD Biosciences).

### Hoechst 33342 staining

Cells were cultured, exposed to olaparib for 24 h, and then fixed with 4% paraformaldehyde for 1 h at room temperature, and stained with Hoechst 33342 in the dark for 30 min. Images were acquired under a Leica MD2700M fluorescence microscope (German).

### Comet assay

Comet assays were performed as described elsewhere [11]. The Comet Assay kit (Trevigen Inc., Gaithersburg, MD, USA) was used under alkalic conditions according to the manufacturer’s specifications. Comets were visualized using a Leica MD2700M fluorescence microscope (German). The tail moments (TMs) of comets were scored using CASP software.

### DR-GFP for HR assay

Cells were transfected the direct-repeat (DR)-GFP plasmid (Addgene, Watertown, MA, USA) by TransIn EL Transfection Reagent (TransGen Biotech, Beijing, China), and the stably expressing the DR-GFP construct were required by G418 screening, and subsequently transiently transfected with pCBASce expression vector (Addgene, Watertown, MA, USA) using TransIn EL Transfection Reagent. GFP signal was assayed 48-h post-transfection on a FACSVantage SE system (BD Biosciences).

### Immunofluorescence

Immunofluorescence were performed as described elsewhere [11, 15]. Briefly, Cells were fixed, permeablized, washed, blocked, and then primary antibodies were applied overnight at 4 °C, and secondary antibodies were applied for 60 min at room temperature. Finally, the cells were washed three times in PBS, and the DNA was stained using DAPI (Sigma-Aldrich, St. Louis, MO, USA) at 5 ng/ml. The slides were observed under a Leica MD2700M fluorescence microscope (German).

### Western blotting and coimmunoprecipitation

Total protein extracts from cells were prepared using RIPA buffer (Beyotime Institute of Biotechnology, Nantong, China). Proteins were fractionated in SDS–polyacrylamide gels, transferred to polyvinylidene fluoride (Millipore, Billerica, MA, USA), and western blotting were performed by using the appropriate antibody. Antibody/antigen complexes were detected using ECL (Western Bright Sirius; Advansta, Inc., Menlo Park, CA, USA) and images were acquired using an enhanced chemifluorescence detection system (Amersham Biosciences, Piscataway, NJ, USA) under the room temperature.

For coimmunoprecipitation, Total protein lysates (0.5 mg) were incubated with 4 μg of specific anti-NFBD1 antibody overnight at 4 °C. NFBD1 immunocomplexes were captured with SureBeadsTM Magnetic Beads (Bio-Rad, Hercules, CA, USA) for 1 h at 4 °C. The resulting immunocomplexes were collected by centrifugation, were washed, boiled in SDS sample buffer, loaded on an SDS–polyacrylamide gel. Proteins were analysed by western blotting using standard methods and detected as described above.

### Xenograft experiments

All animal husbandry and experiments were performed under a protocol approved by Institutional Animal Care Committee at Chongqing Medical University. CNE1 cells (6.0 × 10^6^) in 0.2 ml of growth medium were subcutaneously injected into the axilla of the Balb/c nude mice. Two days after transplantation success (the diameter of tumor was approximately 5 mm), mice were treated daily with either vehicle or 50 mg/kg bodyweight of olaparib intraperitoneally five days a week. Tumor size was measured every week and tumor volume = 1/2 × length × width^2^. Mice were killed after 42 days after cell injection, and then the tumors in the left and right axillary region were excised and weighed.

### Statistics

Statistical comparison of mean values was performed using ANOVA, rank sum test (non-parametric statistics) or chi-square (χ2) test. Differences with a *P*-value of < 0.05 were considered statistically significant.

## Results

### Lentivirus-mediated shRNA inhibited NFBD1 mRNA and protein expression in NPC cells

**Fig. 1:**
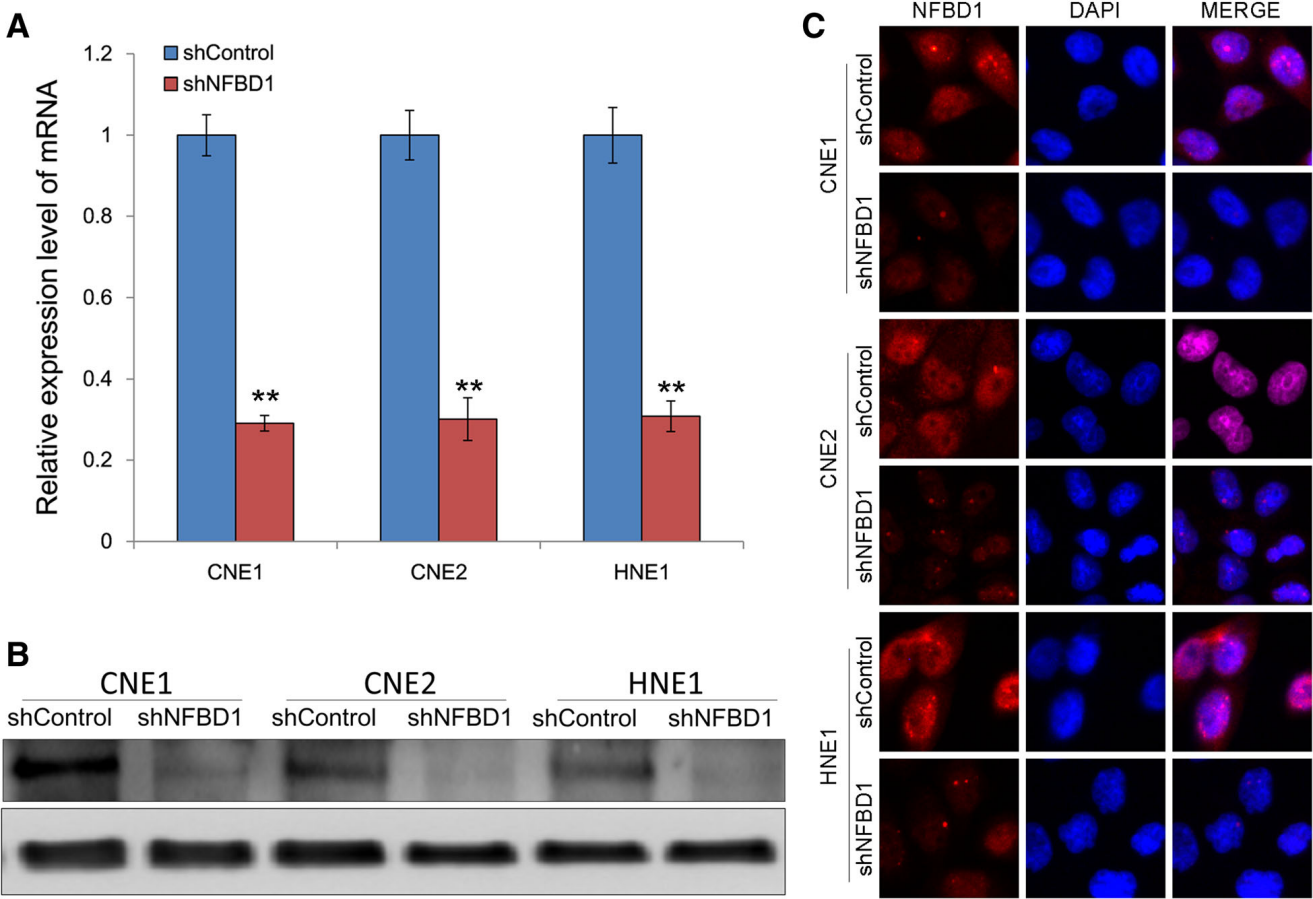
Knockdown of endogenous NFBD1 using lentivirus-mediated shRNA. The lentiviral expressing shNFBD1 and shControl were transfected into CNE1, CNE2 and HNE1 cells. The stable NFBD1-deficiency cells were obtained under puromycin (1 μg/ml). (**a**) Effects of constructed lentiviral on the expression of NFBD1 mRNA were determined by qRT-PCR. The relative expression level of NFBD1 mRNA was significantly downregulated in NFBD1-shRNA group, *P* < 0.01 compared with shControl group. Effects of constructed lentiviral on the expression of NFBD1 protein were determined by western blotting (**b**) and immunofluorescence (**c**). **P < 0.01

The lentiviral expressing NFBD1 shRNA and control shRNA were transfected into NPC cells. The transfected cells of stable expression shNFBD1 and shControl were obtained under puromycin (1 μg/ml). NFBD1 downregulation was confirmed by qRT-PCR and western blotting analysis (Fig. 1a and b). In addition, immunofluorescence also revealed corresponding decrease in the protein levels (Fig. 1c). These results demonstrated that the lentivirus-mediated shRNA targeting NFBD1 effectively knocked down NFBD1 expression at both mRNA and protein levels in the NPC cells.

### Loss of NFBD1 enhances NPC cells to be sensitive to PARP inhibitor olaparib

**Fig. 2:**
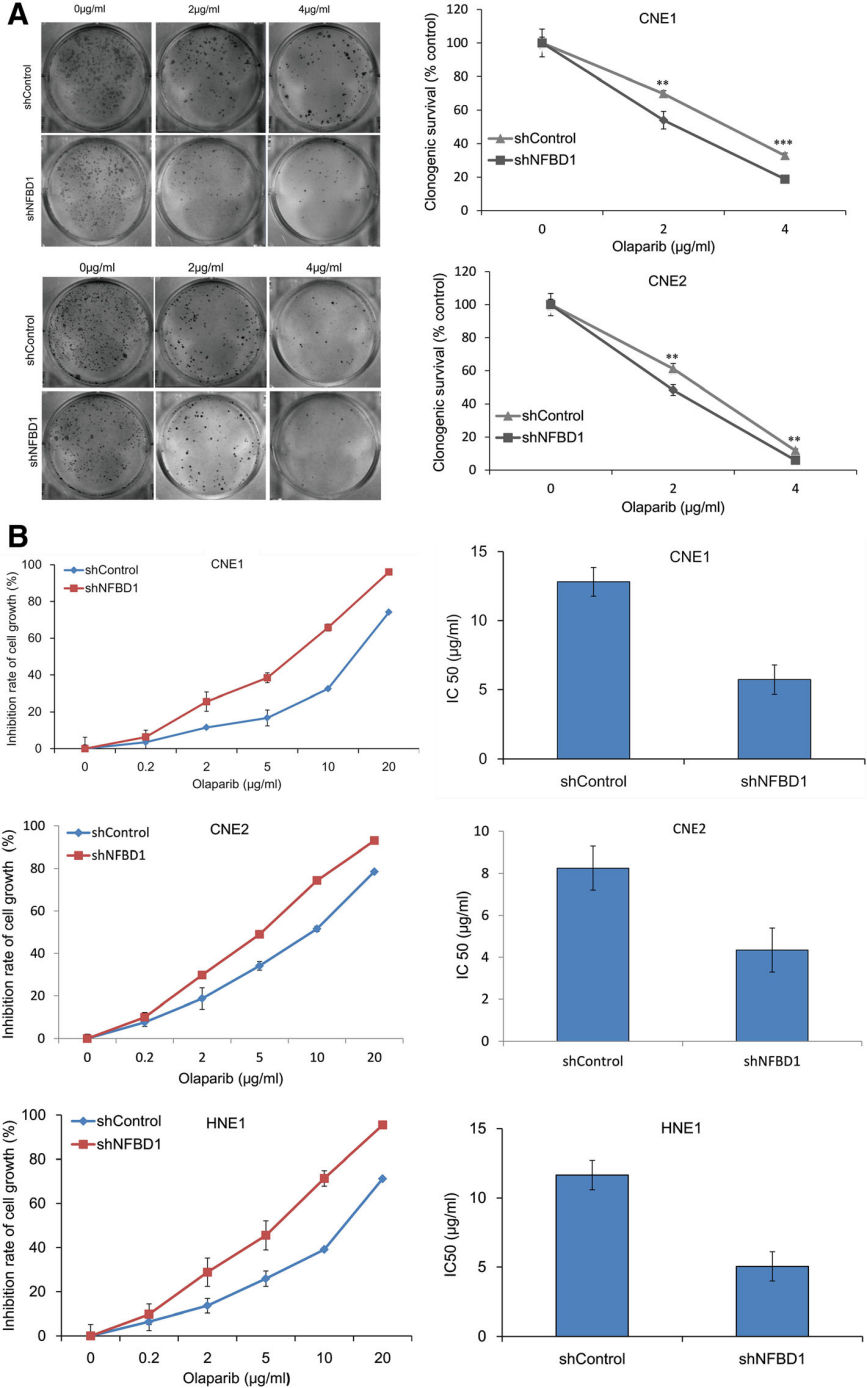
Knockdown of NFBD1 enhances NPC cells to be sensitive to PARP inhibitor olaparib. (**a**) Cells were plated at low density, irradiated and colonies counted after 10–14 days. Results are normalized for effects of NFBD1 and fitted to a standard linear quadratic model. (**b**) NFBD1 knockdown enhanced the cytotoxicity to olaparib in a dose-dependent manner. shControl and shNFBD1 cells were treated with various concentrations of olaparib for 24 h. Cell viability was measured using MTS assays. The data are presented as the mean ± SD of triplicate determinations. IC50 values are presented as the mean ± SD of triplicate determinations. **P < 0.01 and ***P < 0.001

To evaluate the role of NFBD1 in the response of NPC cells to PARP inhibitor-induced DSBs, clonogenic assays were also performed to determine the effects of the NFBD1 knockdown on the sensitivity of NPC cells to olaparib. NPC cells with stable NFBD1 knockdown were markedly sensitive to olaparib compared to control cells (Fig. 2a). In proliferation assays, silencing NFBD1 significantly increased the sensitivity upon olaparib treatment compared to controls, furthermore, NFBD1 knockdown resulted in a marked decrease of IC50 values (Fig. 2b).

### NFBD1 participates in the regulation of olaparib-induced cell cycle distribution and G2/M checkpoint activity

**Fig. 3:**
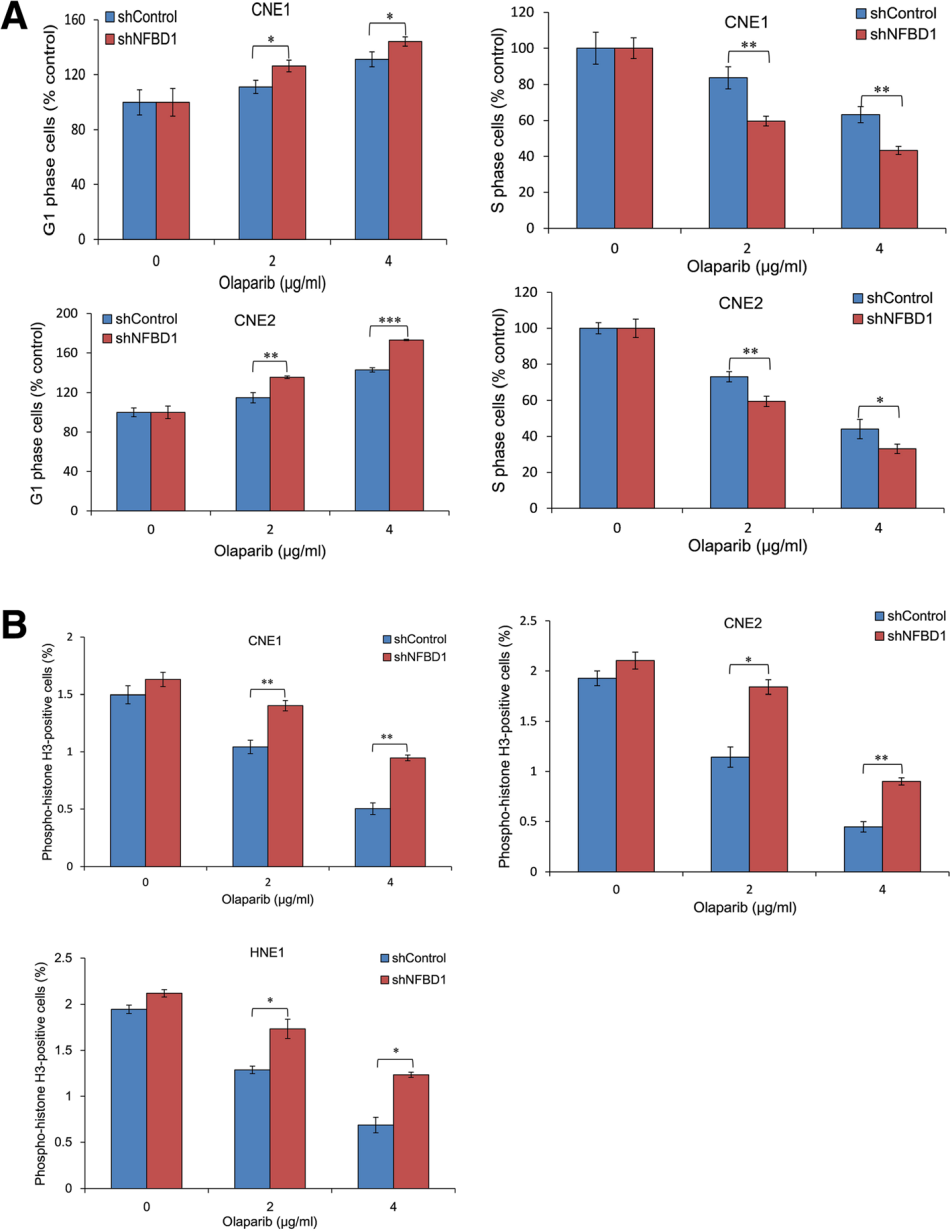
The effects of olaparib on cell cycle distribution. NPC cells were treated with various concentrations of olaparib for 24 h, (**a**) the cell cycle distribution was determined by FCM, and (**b**) mitotic cells were determined by phospho-histone H3 staining and FCM. Data shown are mean ± SD from three independent experiments. *P < 0.05**, *P* < 0.01 and ***P < 0.001

The sensitivity to olaparib suggests an important role in responding to DNA damage; therefore we next tested whether NFBD1 loss could change olaparib-induced cell cycle distribution. shControl and shNFBD1 cells were exposed to olaparib and the cell cycle distribution was determined by flow cytometry (FCM). Control cells exhibited a decrease in the rate of G1 phase, whereas an increase in the rate of S phase compared with shNFBD1 cells (Fig. 3a). This demonstrates a role for NFBD1 in regulating cell cycle progression after DNA damage. We next examined the integrity of the G2/M checkpoint. Cells were exposed to olaparib and labelled with an anti-phospho-histone H3 antibody as a marker of mitotic cells. A clear reduction in phospho-H3-positive cells was observed in the shControl cells after olaparib treatment, whereas a significant number of the cells lacking NFBD1 entered mitosis (Fig. 3b), indicative of a defect in the ability to arrest the cell cycle in G2 phase.

### Deficiency in NFBD1 causes defective DSB repair and confers enhanced apoptosis to the PARP inhibitor olaparib

**Fig. 4:**
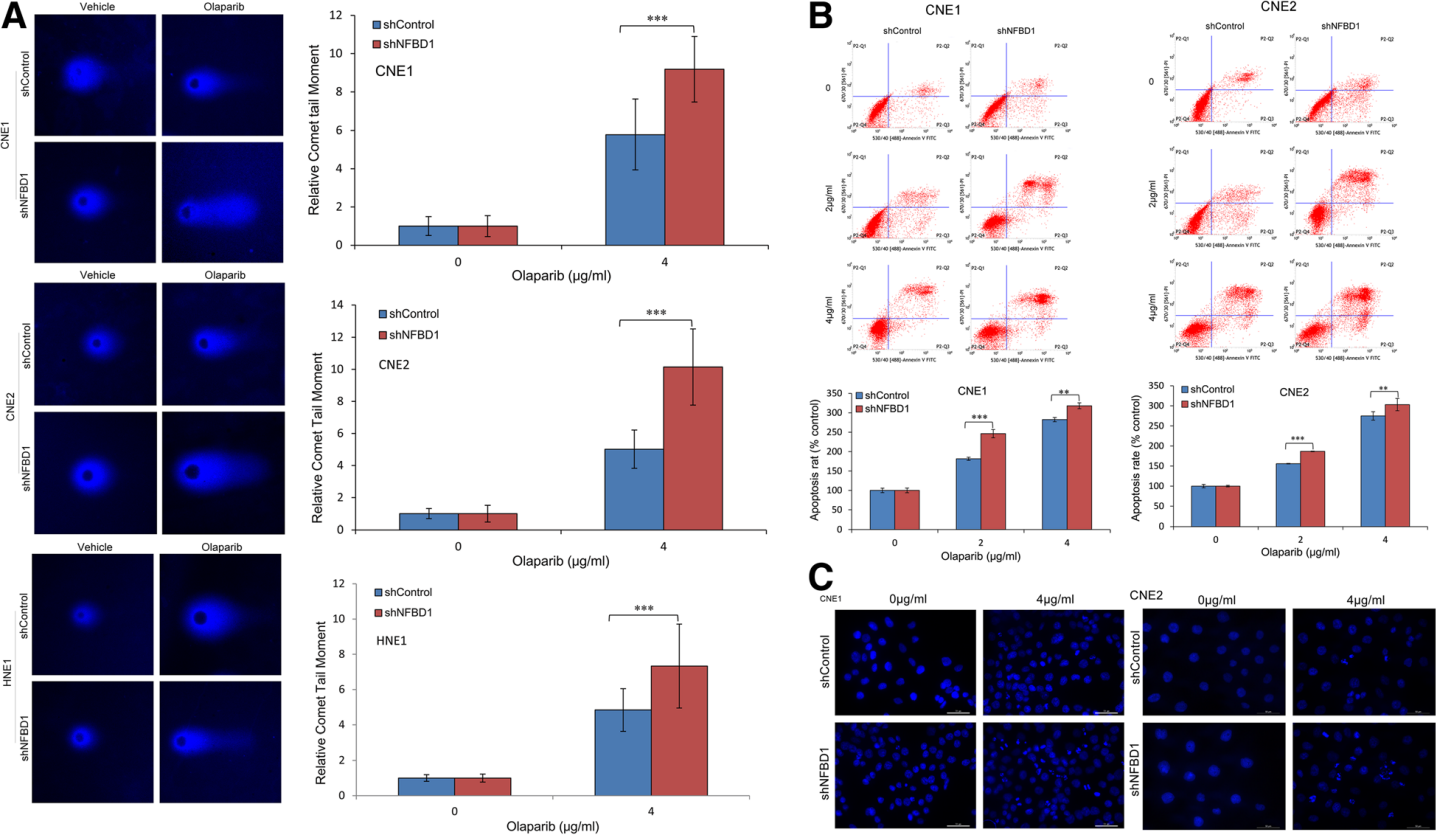
Deficiency in NFBD1 causes defective DSB repair and confers enhanced apoptosis to the PARP inhibitor olaparib **(a)** The repair of DNA damage was detected by comet assay. Tail moments were measured NPC cells treated with 4 μg/ml olaparib for 24 h, and the comet tail moment of 75 cells for each condition was quantified by CASP software and normalized to that of treatment with vehicle. Representative pictures are shown. NPC cells were treated with olaparib for 24 h, the apoptosis was determined by FCM **(b)** and hoechst 33342 staining **(c)**. **P < 0.01 and ***P < 0.001

To determine whether NFBD1 knockdown affects DNA repair post olaparib treatment, we measured the persistence of DNA damage in olaparib-treated NPC cells utilizing comet assay (Fig. 4a). Olaparib treatment induced DSBs, visibly by increased DNA mobility or comet tail. The cells lacking NFBD1 revealed significantly increased the intensity and length of comet tails after olaparib treatment compared with control cells. Considering the notion that cells can undergo apoptosis when DNA damage is irreparable, therefore, to examine whether downregulation of NFBD1 following olaparib treatment can induce apoptosis, NPC cells were exposed to olaparib, and determined the apoptosis rate using FCM (Fig. 4b). Olaparib potently induced apoptosis in CNE1 and CNE2 cells in a dose-dependent manner, furthermore, NFBD1 knockdown significantly enhanced olaparib-induced apoptosis. To further confirm the above result, we performed Hoechst 33342 staining. Consistent with our FCM results, the shRNA group demonstrated a greater number of fragmented nuclei and nuclear shrinkage compared with the shControl group following olaparib treatment (Fig. 4c).

### Silencing NFBD1 abrogates olaparib-induced formation of γ-H2AX and Rad51 foci

**Fig. 5:**
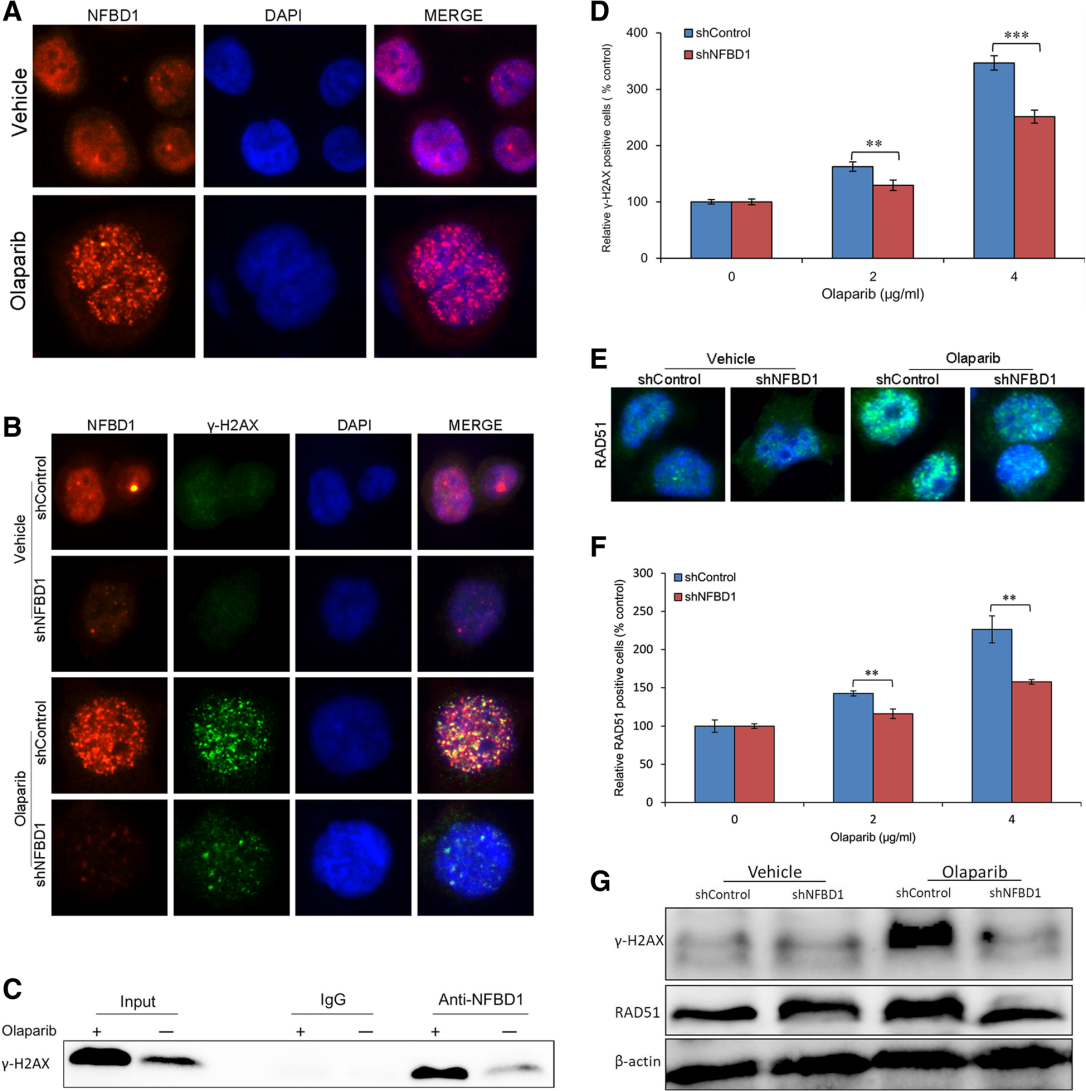
NFBD1 regulates olaparib-induced formation of γ-H2AX and Rad51 foci (**a**) Olaparib-induced NFBD1 foci formation. Inhibition of NFBD1 results in defective γ-H2AX foci formation after olaparib exposure, cells were exposed to olaparib for 24 h, and the γ-H2AX foci formation was detected by immunofluorescence (**b**) and the γ-H2AX positive cells were determined by FCM (**d**). (**c**) Association of NFBD1 with γ-H2AX. Coimmunoprecipitation was done with the antibody indicated or a non-specific, species-matched IgG control. Silencing NFBD1 inhibits RAD51 foci formation, and the RAD51 foci formation was determined by immunofluorescence (**e**) and the RAD51 positive cells was detected by FCM (**f**). (**g**) Cells were untreated or 4 μg/ml olaparib for 24 h, and subjected to western blotting analysis with indicated antibodies. Representative blots were shown with β-actin as loading control. **P < 0.01 and ***P < 0.001

To assess whether NFBD1 could localize to sites of damage, cells were treated with olaparib and stained with anti-NFBD1 antibody. At the level of single nuclei, olaparib induced punctated and distinct NFBD1 foci (Fig. 5a). A proportion of untreated cells also contained NFBD1 foci, indicating that NFBD1 may be responding to endogenous damage or replication stress. As NFBD1 controls the phosphorylation of several checkpoint-responsive proteins, we sought to examine whether it might have a role in H2AX phosphorylation. Silencing NFBD1 significantly affected the phosphorylation of H2AX after olaparib treatment and the formation of γ-H2AX foci (Fig. 5b), the same result was observed in FCM and western blotting analysis (Fig. 5d and g). Furthermore, NFBD1 foci also significantly co-localized with γ-H2AX foci (Fig. 5b and c). Thus NFBD1 was necessary for H2AX phosphorylation and foci formation after olaparib treatment. Because loss of NFBD1 enhances NPC cells to be sensitive to PARP inhibitor olaparib, and the formation of nuclear Rad51 foci is known as the key step of HRR, we ascertained whether NFBD1 had effects on Rad51-mediated repair of DSBs. As hypothesized, immunofluorescence analysis showed that RAD51 foci formation in response to olaparib was greatly inhibited in NFBD1 loss cells (Fig. 5e), and FCM analysis indicated that more RAD51-positive cells were consistently observed after olaparib treatment in the shControl group than that in shNFBD1 group (Fig. 5f). The same result was observed in western blotting analysis (Fig. 5g).

### Loss of NFBD1 disrupts olaparib-induced homologous recombination repair

**Fig. 6:**
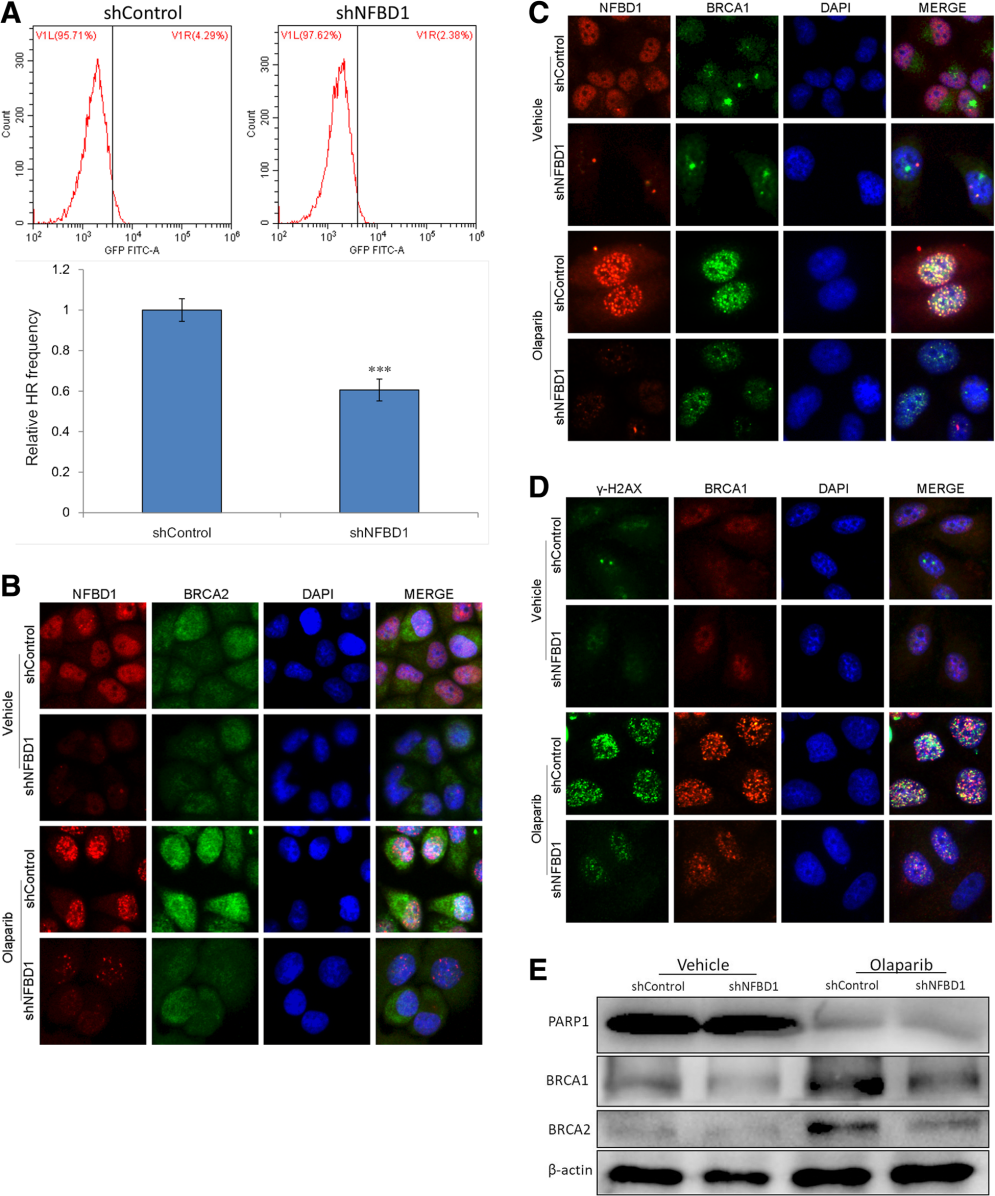
Loss of NFBD1 causes HRR deficiency (**a)** Cells stably transfected with a DR-GFP reporter construct express GFP were transfection of I-SceI endonuclease expression vector. Extent of HR was analyzed by FCM 48 h after transfection. **(b** and **c**) Inhibition of NFBD1 resulted in defective BRCA1 and BRCA2 foci formation after olaparib exposure. (**d**) Silencing NFBD1 disrupts olaparib-induced BRCA1 interaction with γ-H2AX. (**e**) Cells were treated 4 μg/ml olaparib for 24 h, and subjected to western blotting analysis with indicated antibodies. Representative blots were shown with β-actin as loading control

HRR is a high fidelity, error-free form of DNA repair, and BRCA1 and BRCA2 proteins are critical components in the process of HRR. Cancer cells with deleterious BRCA1/2 mutations are defective in HRR and therefore are hypersensitive to PARP inhibitors [17, 18]. We used direct-repeat (DR)-GFP plasmid to assay the HR pathway, and the HR recombination assay relies on the DR-GFP transfected cells to express GFP, if HR activity is functional. The results clearly show significant decrease in percentage GFP-positive cells with NFBD1 inhibition compared to untreated cells (Fig. 6a). Furthermore, we assess whether silencing NFBD1 could affect olaparib-induced the formation of BRCA1 and BRCA2 foci in nuclei. Olaparib induced a marked increase in BRCA1 and BRCA2 foci in shControl cells but not in shNFBD1 cells (Fig. 6b and c), the same results were observed in western blotting analysis (Fig. 6e), and NFBD1 foci also significantly co-localized with BRCA1 foci (Fig. 6c). In addition, BRCA1 was recruited to the DNA damage sites, and co-localized with γ-H2AX foci following olaparib treatment (Fig. 6d).

### NFBD1 loss results in increased PARP inhibitor sensitivity in vivo

**Fig. 7:**
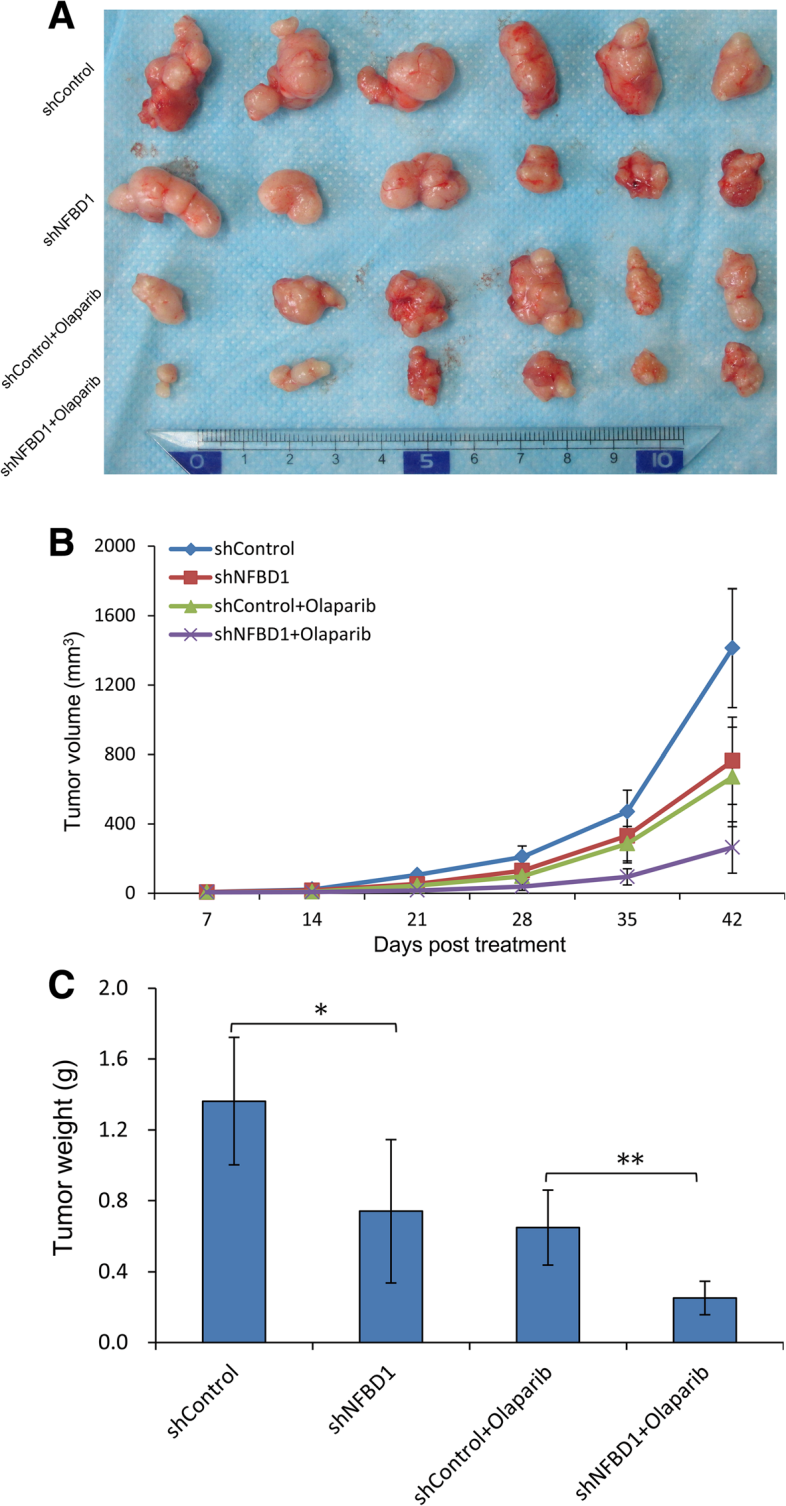
Combination therapy inhibits tumor growth of nasopharyngeal cancer in vivo. (**a** and **b**) Loss of NFBD1 signifcantly reduces xenograft growth after PARP inhibitor olaparib treatment. The tumors were measured every week using a digital caliper. Tumor volume = 1/2 × length × width^2^. (**c**) Tumor weight at 42 days after treatments, *n* = 6 mice per condition. *P < 0.05 and **P < 0.01

To assess the therapeutic effect of olaparib on NFBD1 depleted cells in vivo, we investigated the ability of olaparib to suppress the growth of NFBD1-depleted CNE1 cells derived xenograft tumor. shControl or shNFBD1 cells were subcutaneously grafted into balb/c nude mice. Two days after transplantation success, mice were treated with olaparib or vehicle. Six weeks post-treatment, mice were sacrificed and tumor volume measured and quantified. Our results showed that either downregulation of NFBD1 or olaparib alone resulted in significantly smaller tumor than untreated xenografts, however, the combination of downregulated NFBD1 and olaparib resulted in significantly smaller tumors as compared with untreated controls or to tumors treated with downregulated NFBD1 or olaparib alone (Fig. 7a–c). Thus, the results suggested that silencing NFBD1 enhanced the response of NPC cells to olaparib and resulted in tumor growth inhibition in vivo.

## Discussion

Cells are continuously exposed to exogenous and endogenous agents that lead to DNA damage, which, if not repaired effectively and efficiently, can result to genomic instability or cell death [19]. The HRR pathway is an especially important DNA repair pathway in this respect. HR-deficiency is observed in some cancers, such as breast cancer and ovarian cancer. Interestingly, tumors with defective HR, including BRCA1/2 mutation cancers, exhibit particular sensitivity to PARP inhibitors [20–23]. However, the use of PARP inhibitors is limited to cancer patients with BRCA1/2 mutations, which represent only a small subset of cases. Therefore, new therapeutic strategies and approaches are urgently needed to make broader use of PARP inhibitors for the majority of other tumor cases.

Conceptually, PARP inhibitors can selectively augment DNA damage in tumor with HR-deficiency compared with normal tissues. As implicated by previous studies, NFBD1 has important roles in DDR, involving a complex network of signaling pathways that regulates cell cycle checkpoints, DNA repair and cell death [11, 15, 24–27]. Our studies showed that NFBD1 protein is highly expressed in nasopharyngeal carcinoma tissues, and shRNA targeting NFBD1 enhanced the radiosensitivity of CNE1 cells and impaired DNA damage-induced RAD51 foci formation [11, 15, 16]. This suggested that the rationale for the utility of olaparib is based upon the notion that silencing NFBD1 disturbs the HRR pathway required for DNA damage repair following olaparib treatment. In this study, silencing NFBD1 significantly enhanced olaparib-induced apoptosis and growth inhibition of NPC cells. More importantly, NFBD1 knockdown can delay olaparib-induced DNA damage repair, impair cell cycle checkpoint activity, and enhance the sensitivity of NPC cells to olaparib. Xenografts models showed that silencing NFBD1 significantly enhanced the anti-tumor activity of olaparib, leading to tumor growth inhibition of the combination therapy. Mechanistic studies revealed that silencing NFBD1 disturbed the HRR pathway through impairing the foci formation of olaparib-induced BRCA1, BRCA2 and RAD51. These findings provide a molecular basis of using PARP inhibitors to potentiate treatment of nasopharyngeal carcinoma with NFBD1-deficiency.

It has been argued that cell cycle checkpoint arrest, although important for maintaining genomic stability post DNA damage, makes a less significant contribution to survival. However, when cell cycle checkpoint inactivation is combined with defective DSBs repair, the impact is more than additive, consistent with the notion that cell cycle checkpoint arrest enhances the opportunity for DSBs repair [28]. In our studies, olaparib treatment led to a significant increase in G1 and M phase cell population and a corresponding decrease in S phase for NFBD1-deficiency NPC cell lines, indicative of a defect in the G1/S and G2/ M checkpoint. Furthermore, the comet assay showed that silencing NFBD1 significantly increased olaparib-inducing DNA damage. From these data, silencing NFBD1 enhanced the sensitivity of NPC cells to olaparib through impairing cell cycle checkpoint activity and improving DNA damage.

On the basis of our findings, we propose that the enhanced sensitivity of NFBD1-deficiency cells to oalaprib is largely due to defect of HRR pathway. In the case of DNA damage, γ-H2AX is ATM-dependent, which is involved in the amplification step required for optimal checkpoint response in the DDR [29, 30]. It is evident that a network of interactions is initiated around γ-H2AX, which recruits and maintains many DDR proteins at sites of DSBs [31–35]. We found that olaparib induced punctated and distinct NFBD1 and γ-H2AX foci, and BRCA1 was also recruited to the DNA damage sites and colocalizated with γ-H2AX. However, knockdown of NFBD1 greatly reduced olaparib-induced formation of γ-H2AX foci, moreover, our colocalization studies indicated NFBD1 foci extensively overlap with γ-H2AX foci, suggesting that NFBD1 was required for the formation of olaparib-induced γ-H2AX foci. In addition, NFBD1- deficient cells revealed a decrease BRCA1, BRCA2 and RAD51 foci relative to controls, which indicated the use of NFBD1 loss combination with olaparib may directly inhibit HR-mediated DNA repair through impairing the foci formation of BRCA1, BRCA2 and RAD51. The impairment of DDR proteins foci formation could be due to overall protein level is decreased, and/or HR proteins cannot be recruited to DSB sites. Clearly, the western blotting shows that the depletion of NFBD1 significantly reduces the protein level of BRCA1, BRCA2, and RAD51. Therefore, these results indicated that downregulation of NFBD1 can inhibited the amplification of the olaparib-induced DNA damage signal, decreased the levels of DDR proteins, and failed to accumulate and retain DDR proteins at the sites of DNA damage, which leaded to defective HRR activation following olaparib treatment.

## Conclusions

We report here that NFBD1 knockdown combination with olaparib can enhance the sensitivity of olaparib in NPC cells. Silencing NFBD1 also inhibit the amplification of the olaparib-induced DNA damage signal, and fail to accumulation and retain DDR proteins at the sites of DNA damage, which leads to defective checkpoint activation following DNA damage. Furthermore, silencing NFBD1 inhibits the HRR pathway activity by impairing the foci formation of olaparib inducing BRCA1, BRCA2 and RAD51. Our studies provide compelling evidence that combining depletion of NFBD1 and olaparib represents a rational strategy for the treatment of patients with NPC.

## Abbreviations

BER: Base excision repair
DDR: DNA damage response
DSBs: DNA double-strand breaks
FCM: Flow cytometry
HR: Homologous recombination
HRR: Homologous recombination repair
NHEJ: Non-homologous end-joining
NPC: Nasopharyngeal carcinoma
PARP: Poly (ADP-ribose) polymerase
qRT-PCR: Real-time quantitative RT-PCR
SSB: Single-strand break
